# Ozone Therapy in the Management of Persistent Radiation-Induced Rectal Bleeding in Prostate Cancer Patients

**DOI:** 10.1155/2015/480369

**Published:** 2015-08-18

**Authors:** Bernardino Clavo, Norberto Santana-Rodriguez, Pedro Llontop, Dominga Gutierrez, Daniel Ceballos, Charlin Méndez, Gloria Rovira, Gerardo Suarez, Dolores Rey-Baltar, Laura Garcia-Cabrera, Gregorio Martínez-Sánchez, Dolores Fiuza

**Affiliations:** ^1^Radiation Oncology, Dr. Negrin University Hospital, 35.010 Las Palmas, Spain; ^2^Chronic Pain, Dr. Negrin University Hospital, 35.010 Las Palmas, Spain; ^3^Experimental Surgery-Research Unit, Dr. Negrin University Hospital, 35.010 Las Palmas, Spain; ^4^Canary Islands Institute for Cancer Research (ICIC), 35.010 Las Palmas, Spain; ^5^Spanish Group for Clinical Research in Radiation Oncology (GICOR), Madrid, Spain; ^6^Gastroenterology, Dr. Negrin University Hospital, 35.010 Las Palmas, Spain; ^7^Ozone Therapy Unit, Quiron Hospital, 08.023 Barcelona, Spain; ^8^Medical Center Beauty Benefit-San Biagio di Osimo, Osimo, 60.027 Ancona, Italy

## Abstract

*Introduction*. Persistent radiation-induced proctitis and rectal bleeding are debilitating complications with limited therapeutic options. We present our experience with ozone therapy in the management of such refractory rectal bleeding. *Methods*. Patients (*n* = 12) previously irradiated for prostate cancer with persistent or severe rectal bleeding without response to conventional treatment were enrolled to receive ozone therapy via rectal insufflations and/or topical application of ozonized-oil. Ten (83%) patients had Grade 3 or Grade 4 toxicity. Median follow-up after ozone therapy was 104 months (range: 52–119). *Results*. Following ozone therapy, the median grade of toxicity improved from 3 to 1 (*p* < 0.001) and the number of endoscopy treatments from 37 to 4 (*p* = 0.032). Hemoglobin levels changed from 11.1 (7–14) g/dL to 13 (10–15) g/dL, before and after ozone therapy, respectively (*p* = 0.008). Ozone therapy was well tolerated and no adverse effects were noted, except soft and temporary flatulence for some hours after each session. *Conclusions*. Ozone therapy was effective in radiation-induced rectal bleeding in prostate cancer patients without serious adverse events. It proved useful in the management of rectal bleeding and merits further evaluation.

## 1. Introduction

Of the possible complications secondary to radiotherapy (RT) for prostate cancer, rectal bleeding (RB) is one of the most relevant. The incidence and severity of late effects of RT depend on dosimetry factors (total dose of RT, rectal volume exposed to high doses, and 2D versus 3D conformation), fractionation (fractions of RT dose administered each day), technique for radiotherapy delivery (external beam radiotherapy versus brachytherapy), and patient factors (such as previous inflammatory colitis disease, vascular diseases as in diabetes which could adversely affect the healing process, and antiaggregant or anticoagulant treatments which can encourage bleeding [1, 2]). With previous and current, external beam RT, Grade 3 or Grade 4 rectal toxicity has been described as occurring in between 1% [3] and 5% [4] of cases, with this percentage increasing up to 32% at 5 years (if Grade 2 or higher toxicities are included) [4]. With brachytherapy as well, Grade 2 and Grade 1 rectal toxicities at 5% and 1%, respectively, have been described [5]. Hence, radiation proctitis and RB remain relevant side effects following treatment of this tumor.

In a large study of 32465 patients, complications other than incontinence or erectile dysfunction after treatment of localized prostate cancer had a 5-year cumulative incidence of admission-to-hospital of 22%. Most hospital admissions in prostate cancer patients treated surgically were for urinary obstruction whereas, in patients treated with radiotherapy, the highest number of hospital admissions was for radiation proctitis [6]. Mild symptoms of radiation proctitis may remit spontaneously (without specific therapy) or with medical treatments involving endoscopy interventions. When RB becomes severe or persistent despite consecutive endoscopy procedures, treatment options become limited and as many as 5% of patients can become transfusion-dependent [2]. Hyperbaric oxygen (HBO) is the most-documented alternative therapy [7]. However, it needs extensive facilities since the equipment is cumbersome, it is of limited availability in most centers, and it has potential side effects such as claustrophobia, headache, vomiting, convulsions, and risk of barotrauma (ears, sinuses, and lung) [8]. Major surgical intervention is restricted, usually, to the most severe or life-threatening conditions because of the potential morbidity and mortality [9].

Although their mechanisms of actions are different, several effects of ozone therapy (O_3_T) have been observed that are similar to that associated with HBO [10]. A century ago a description was published on how local O_3_T application could improve radiation-induced side effects [11], and rectal O_3_ insufflations were described a couple of decades later [10]. We have published successful management of several radiation-induced toxicities using O_3_T [12–15]. The aim of this current report is to present the effect of ozone therapy in the management of persistent radiation-induced RB in prostate cancer patients.

## 2. Materials and Methods

Patients (*n* = 12) with persistent RB were evaluated and underwent O_3_T between April 2004 and October 2008. Median age was 70 years (range: 64–77). O_3_T has been used over several years in our hospital (Chronic Pain Unit and Radiation Oncology Department) for complementary treatment for wound healing, ischemic syndromes, and pain. Without hyperbaric oxygen facilities in our hospital, O_3_T was administered on compassionate grounds in these patients with persistent symptoms despite conventional management. All patients provided informed written consent to the therapy and all procedures conformed to the Helsinki Declaration of 1975. All patients had prostate cancer and had been receiving treatment between the years 2002 and 2008. The patients' data are summarized in Table 1.

Radiation proctitis diagnosis was by endoscopy in all patients and additional rectal biopsy in 5 patients. Prior to O_3_T, all patients were under conservative management including high-fiber diet and laxatives, oral or rectal drugs (sucralfate, mesalazine, or corticosteroid), and/or endoscopy procedures (argon laser, formalin, and coagulation). In the most severe cases, antiaggregating agents were temporarily withdrawn.

Severity of rectal hemorrhage was classified (Table 2) according to the National Cancer Institute Common Terminology Criteria for Adverse Events (CTCAE) grading system (version 4.0) [16].

### 2.1. Ozone Therapy Procedure

Our procedures for O_3_T application in radiation proctitis have been described previously [14] and conform to international guidelines of the “Madrid Declaration on Ozone Therapy” [17]. These consisted of two different techniques: rectal insufflations of O_3_/O_2_ gas mixture (applied in 11 patients) and topical application of ozonized-oil (in 8 patients).

For rectal insufflations, an accurate concentration of O_3_/O_2_ gas mixture (*μ*g/mL: *μ*g of O_3_ per mL of O_2_) was obtained from clinical grade oxygen using a medical ozone generator (Ozonosan Alpha-plus,* Dr. Hänsler GmbH*, Iffezheim, Germany). Standard rectal cannula and 60 mL silicone syringes were used for rectal insufflation. According to patient tolerance to the bowel distension (or bloating) insufflation gas volume was between 150 and 300 mL for each session. We followed the concept “*start low*,* go slow*” [18]. O_3_ concentrations at the first session were 5 *μ*g/mL in 5 patients and 10 *μ*g/mL in 6 patients. The concentration was progressively increased to 20 *μ*g/mL in all patients and increased further to 30 *μ*g/mL in 4 patients. Overall, most sessions were performed at an O_3_ concentration of 20 *μ*g/mL, with an insufflated gas volume of 180–240 mL and with total O_3_ administration between 3600 and 4800 *μ*g of O_3_ in each session. In one patient (patient #5) 2 sessions of 50 *μ*g/mL (and 60–100 mL) were administered at the time of more intense bleeding. Ozone therapy was initially scheduled for 3 times/week. When patients showed clinical improvement (the decrease in the frequency and intensity of the bleeding being maintained), the number of sessions was progressively reduced to 2 sessions and 1 session/week, followed by 2 sessions/month and, finally, 1 session/month for 2-3 additional months. Time spent for each O_3_T session of rectal insufflation was around 15–20 minutes. It is important to note that the production and administration of ozone need to be performed* in situ* in the outpatient clinic because O_3_ as a gas spontaneously decomposes to O_2_ with a half-life of 40 minutes at 20°C [10].

Topical ozonized-oil was applied in 8 patients. Commercial ozonized-oil (*Dr. Hänsler GmbH,* Iffezheim, Germany) was mixed in our hospital's pharmacy with liquid-Vaseline to provide ozonized-oil; concentrations ranged from 14 to 25%. The 25% mixture (5 to 10 mL) was administered 1-2 times/day, initially. When patients showed clinical improvement, the O_3_ concentration and the frequency of administration were progressively reduced. Since the half-life of ozonized-oil can range from months to years (depending on storage temperature), this approach allows self-administration at home and was especially relevant for patients residing a considerable distance away from our center.

Endoscopy requirements were at the discretion of the attending gastroenterologist.

O_3_T was well tolerated and no adverse effects were noted, except soft and temporary flatulence for some hours after each session.

### 2.2. Statistical Analyses

The SPSS software package (version 15 for Windows) was used for all statistical analysis. Nonparametric tests were used for all comparisons because most variables were “nonnormally” distributed. Values are presented as median (range: minimum–maximum). Comparisons of paired data before and after therapy were with two-tailed Wilcoxon signed-rank test and Friedman's test for comparisons of related data at the 3 time-points before O_3_T, after O_3_T, and at final follow-up clinical visit. Correlations were assessed using Spearman's correlation coefficient (rho). Comparisons of categorical values were with the *χ* ^2^ test. Statistical significance was set at *p* < 0.05.

## 3. Results

Median time-lapse between the end of radiotherapy and rectal bleeding was 3 months (range: 0–34). Median time-lapse between the onset of rectal bleeding and commencing O_3_T was 10 months (range: 2–23). Time-lapse between the end of radiotherapy and commencing O_3_T was 16 months (range: 8–36). Median duration of O_3_T was 13 months (range: 3–19). The median number of O_3_T sessions was 38 (range: 14–107).

Half of the patients had needed blood transfusions before O_3_T commencement and 26 blood transfusions had been administered (median: 3; range: 1–11). The lowest hemoglobin value before O_3_T was a median of 7.3 g/dL (range: 5–14). Median hemoglobin value just before O_3_T commencement was 11.1 g/dL (range: 7–14) and 9 patients (75%) were anemic. At the end of O_3_T, only 4 patients (33%) were anemic compared to pre-O_3_T values (*p* = 0.101) and the median hemoglobin value was 13 g/dL (range: 10–15) g/dL (*p* = 0.008).

Before commencing O_3_T, a total number of 37 (median 4; range 0–10) unsuccessful endoscopy treatments had been performed in 8 patients (67%). The endoscopy was reduced to 17 procedures in 4 patients (median 0; range 0–8; *p* = NS) during O_3_T and to 4 procedures in 2 patients during follow-up (median 0; range 0–2; *p* = 0.015). Overall, the change in the number of endoscopy treatments applied was statistically significant (*p* = 0.032).

The median CTCAE toxicity grade was 3 (range: 2–4) before O_3_T; it was reduced to 1 (range: 0-1) by the end of O_3_T (*p* = 0.002) and to 0 (range: 0-1) at the last outpatient clinic follow-up (*p* < 0.002). Overall, the change in CTCAE toxicity grade was statistically significant (*p* < 0.001) (Table 2).

CTCAE toxicity grade before O_3_T was inversely correlated with time-lapse between the end of radiotherapy and rectal bleeding (rho = −0.588, *p* = 0.044) and showed a trend towards a borderline significant direct correlation with O_3_T duration (rho = 0.574, *p* = 0.051) and number of rectal O_3_T sessions (rho = 0.648, *p* = 0.059); that is, higher toxicity grade was related to earlier commencement of RB and longer treatment with O_3_T. Toxicity grade before O_3_T also showed an inverse trend towards a correlation with the lowest hemoglobin levels reached by the patient prior to O_3_T commencement (rho = −0.563, *p* = 0.078); that is, the greater the toxicity grades, the lower the hemoglobin levels.

Duration of O_3_T (rectal insufflations and/or ozonized-oil) was significantly higher in younger patients (rho = 0.602, *p* = 0.038).

Four of the 12 patients (33%) were receiving antiaggregant treatment, and this showed a trend (albeit not statistically significant) towards a correlation with a higher requirement for blood transfusions before O_3_T (rho = 0.606, *p* = 0.063).

Median follow-up after O_3_T was 104 months (range: 52–119). Median follow-up after RT was 136 months (range: 69–149). No patient had died from prostate cancer during follow-up period. Two patients (patient #2 and patient #3) died without evidence of tumor or biochemical relapse at 52 and 65 months after the end of O_3_T. Both patients were under antiaggregant treatment for vascular comorbidities and in both cases death was secondary to persistent and refractory radiation-induced hematuria. Only one patient (patient #6) showed biochemical progression (33 months after the end of RT) with one isolated bone metastasis being noted. This patient was treated with RT for the bone metastasis and started permanent hormonal blockade without further biochemical relapse, or evidence of tumor, at the last follow-up 7 years later.

## 4. Discussion

Of the possible complications secondary to radiotherapy for prostate cancer, radiation proctitis is not unusual, although assessment and quantification of the different symptoms are not easy. We have used the CTCAE toxicity scales focusing on hemorrhagic symptoms. After O_3_T, there was a significant, and clinically relevant, decrease in CTCAE toxicity grade: median grade toxicity was decreased from Grade 3 to Grade 1 before and after O_3_T, with all patients achieving Grade 0 or Grade 1 toxicity status by the end of follow-up. These findings concur with results described with HBO where the improvement achieved was stable (or tended towards further improvement) throughout follow-up [7]. Quality-of-life is an important issue in cancer patients and this is even more relevant for those tumors where a longer survival, as is usually the case with prostate cancer, can be offered. At the end of O_3_T the perception recorded by the patients in our study group was 100% improvement in 7 patients (58%), >75% in 3 patients (25%), and an improvement of around 50% for the other 2 patients.

Proctitis management depends on the severity of symptoms. Grade 1 radiation proctitis usually remits spontaneously without specific therapy within 6 months in about 1 out of 3 patients [8]. First-level treatment includes laxatives, rectal or oral anti-inflammatory drugs (mesalazine, sulfasalazine, or glucocorticosteroids), and sucralfate [19] or sodium butyrate enemas [20]. Second-level treatment includes coagulation via endoscopy using bipolar or heater probes [21] or argon plasma and formaldehyde application for the more persistent symptoms [22, 23]. Third-level treatment includes HBO [24]. Its beneficial effect has been confirmed by a double-blind randomized clinical trial [7] but it is not readily available in most hospitals. Finally, the last option is surgery, which is reserved for the most adverse clinical conditions because it is often associated with higher morbidity; that is, radiation-induced damage leading to RB is also associated with potential altered healing after surgery [2]. Several treatment-algorithms for RB secondary to chronic radiation proctopathy have been proposed [2, 25]. Over the years, we have used several of these first- and/or second-level treatments in our patients.

The systemic effects of O_3_T augur well for its use in the management of chronic radiation proctitis. Chronic radiation proctitis results from lesions in the connective tissues and blood vessels, leading to chronic ischemia and persistent prooxidative status with/without additional inflammation [26]. O_3_T can improve hemorheological parameters [27], blood flow, and oxygenation in hypoxic tissues [15, 28, 29] and can induce a beneficial modulation of the immune-inflammatory response [10]. Additionally, these vascular effects of O_3_T widen its usefulness in combination with HBO (if/when available) because the high arterial pO_2_ levels obtained by HBO tend to induce vasoconstriction while the O_3_T mitigates this effect.

Ozone does not act on specific receptors; its main mechanism of action is indirect, that is, by the production of a “controlled and moderate” oxidative stress which induces subsequent adaptive responses [30]. O_3_ reacts rapidly with antioxidants and polyunsaturated fatty acids (PUFA). The results are lipid oxidation products and induction of intracellular second messengers, of which the most important are hydrogen peroxide (H_2_O_2_) and alkenals (mainly 4-hydroxynonenal, 4-HNE) [31]. These second messengers lead to the activation of nuclear transcriptional factors such as nuclear factor- (erythroid-derived 2) like 2 (Nrf2) which results in the transcription of antioxidant response elements (ARE) and subsequent production of antioxidant enzymes including superoxide dismutase, glutathione-peroxidase, heat shock proteins (HSP-70), and heme oxygenase-1 (HO-1) [31–33]. Additionally, Nrf2 can lead to suppression of nuclear factor kappa B (NF*κ*B) which has a proinflammatory effect. By producing a controlled oxidative stress, O_3_T can modulate the immune response by suppressing NF*κ*B and inducing other nuclear transcription factors such as nuclear factor of activated T-cells (NFAT) and activated protein-1 (AP-1), as well as further modulation of interferons and interleukins [34].

Ozone does not have a linear dose-response relationship but follows the concept of “hormesis” in which a dose high enough to induce an adaptive response needs to be used but not sufficiently high to be harmful [18]. The molecular mechanisms of action of O_3_ have been described in recent reviews [10, 18, 30, 32, 33, 35]. Chronic ischemia, oxidative stress, and inflammation are usually present in tissues in the course of radiation-induced side effects. As mentioned above, O_3_T can modulate these factors. Hence, O_3_T could be a valuable adjuvant in the management of radiation proctitis as has been documented for the use of corticosteroids (anti-inflammatory effect), vitamin E and vitamin C [36], or vitamin E with pentoxifylline [37] (antioxidant effect) or acting against ischemia/hypoxia using HBO or pentoxifylline [7, 37]. Indeed, O_3_T could enhance the effect of HBO by decreasing HBO-associated vasoconstriction secondary to hyperoxia and, as such, enhancing the effects of other medical or endoscopic treatments deployed in these patients.

Some reports have indicated success of local O_3_T applications in radiation-induced toxicity [11–14]. In our study group, rectal O_3_ insufflations provided systemic and local effect and topical application of ozonated-oil provided an additional local effect. The ozonated-oil does not penetrate through the mucous membranes but, instead, reacts with PUFA to induce cellular production of hydrogen peroxide and alkenals which act as second messengers in improving wound healing [38, 39]. The beneficial effect of ozonated-oil in radiation proctitis is currently supported by a preclinical study where ozonated-oil decreased macroscopic and pathological findings of acute radiation proctitis in rats [40]. These findings concur with a similar beneficial effect described in bisphosphonate-induced osteonecrosis of the jaw in cancer patients [41, 42]. The last interesting property of the local application of O_3_T for the management of RB and radiation proctitis is its bactericidal properties [38, 39], an effect that has been exploited in the treatment of public drinking water in many cities. The use of antianaerobic antibiotic metronidazole in radiation proctitis follows this rationale [43].

Clarke et al. [7] reviewed randomized studies using HBO in 120 patients for the treatment of radiation-induced proctitis. Many of the patients included in the studies had received HBO for nonbleeding symptoms. Of note was that only 14 patients were male, despite the study representing one of the largest works in the field. Our results compare favorably even though all our patients had been treated for rectal bleeding, symptoms that imply more advanced toxicity (83% had Grade 3 or Grade 4 toxicity) and, consequently, poorer outcomes expected. Our patients had had a high requirement for endoscopy treatments and blood transfusions. For example, therapeutic endoscopy had been received by 8 of the 12 patients (67%) in our study. Mayer et al. described a group of 10 prostate cancer patients with radiation proctitis treated with HBO. The patients represented 60% with Grade 3 and none with Grade 4 toxicity, and only 30% had undergone previous treatment with laser coagulation [24].

The number of O_3_T sessions required for successful treatment of radiation-induced RB depends on the individual patient. In our study, the median number of treatment sessions was 38 (range: 14–107) and is similar to reports using HBO. The review by Clarke et al. [7] of randomized studies showed a median number between 20 and 80 HBO sessions, while the study by Mayer et al. [24] reported a median of 30 HBO sessions (range: 13–60).

Our study has several limitations. This is a noncontrolled study and, as such, the existence of some bias or subjective influence cannot be excluded. Similarly, there is the uncertainty regarding the real impact on the clinical evolution of the symptoms resulting from not having the O_3_T administered. However, 83% of the patients in our study had persistent Grade 3 or Grade 4 toxicity despite conventional treatment. This would have presented questionable ethics of not offering an option (potentially therapeutic or palliative) that was available in our center, an experimental study of radiation proctitis with a control group having described the beneficial effect of the ozone treatment [40]. Another important limitation of the study is the limited sample size. However, many studies in this field have similar sample sizes, and most of the studies with larger sample sizes had enrolled males as well as females and with different tumors and, as such, with different radiation doses. All patients in our study were irradiated for prostate carcinoma at similar high doses of radiation (70–74 Gy), that is, higher than in other pelvic tumors (gynecological, bladder, or rectal tumors).

Finally, it needs to be highlighted in our study that the median follow-up after O_3_T was 104 months (range: 52–119), which is considerably longer than the median follow-up reported in other studies: 12 months (range: 8–27) [24] or 2 years (3–60 months) [7]. The significant and clinically relevant decrease in the grade of toxicity following O_3_T was maintained for this protracted follow-up period of 104 months and is of considerable benefit to the patient.

In conclusion, our results show a significant, and clinically relevant, effect of ozone therapy in the management of radiation-induced rectal bleeding. Following ozone therapy, the requirements for blood transfusions were significantly decreased as was the need for endoscopic procedures. The grades of toxicity (according to the CTCAE scale) were also reduced. These effects were maintained over the course of protracted follow-up, and no adverse impact on survival was noted. We believe the local application of ozone therapy can be useful as adjuvant treatment in managing radiation proctitis and, as such, merits further evaluation in randomized clinical trials.

## Figures and Tables

**Table 1:** Clinical characteristics of the patients in the study prior to initiation of ozone therapy.

Patient	Age	LHB	HB	Blood transfusion	Endoscopic treatments	Toxicity score
#1	73	7.4	8	5	3	4
#2	74	6.6	7	4	5	3
#3	69	5.6	7	3	4	3
#4^*∗*^	67	5	12	0	0	4
#5	66	7.3	11	1	0	3
#6	64	12.7	14	0	5	3
#7	73	6.1	7	2	5	3
#8	70	11.6	12	0	0	2
#9	69	6.5	8	11	4	4
#10	73	11.8	12	0	0	3
#11	68	NA^*∗∗*^	NA^*∗∗*^	0	10	3
#12	77	14	14	0	1	2

Note: LHB: lowest hemoglobin value (in g/dL) prior to initiation of ozone therapy.

HB: hemoglobin level in g/dL prior to the initiation of ozone therapy.

^*∗*^Patient #4 was a referral for surgery from a different hospital, and details of previous blood transfusion and therapeutic endoscopy were not available.

^*∗∗*^NA: patient #11 did not have anemia, but the precise hemoglobin value had not been recorded.

Toxicity grade was according to the National Cancer Institute Common Terminology Criteria for Adverse Events (CTCAE) grading system (version 4.0): available from http://evs.nci.nih.gov/ftp1/CTCAE/CTCAE_4.03_2010-06-14_QuickReference_5x7.pdf.

**Table 2:** Rectal hemorrhage grading according to CTCAE^*∗*^.

Grade	Criteria	Before O_3_T	End O_3_T	Last follow-up
0	No symptoms	0	5	7
1	Mild; intervention not indicated	0	7	5
2	Moderate symptoms; medical intervention or minor cauterization indicated	2	0	0
3	Transfusion, radiologic, endoscopic, or elective operative intervention indicated	7	0	0
4	Life-threatening consequences; urgent intervention indicated	3	0	0

Note: CTCAE toxicity grade was significantly decreased at the end of O_3_T (*p* < 0.002) and at the last follow-up (*p* < 0.002) compared to that before O_3_T. Overall, the change in CTCAE toxicity grade was statistically significant (*p* < 0.001).

^*∗*^According to the National Cancer Institute Common Terminology Criteria for Adverse Events (CTCAE) grading system (version 4.0): available from http://evs.nci.nih.gov/ftp1/CTCAE/CTCAE_4.03_2010-06-14_QuickReference_5x7.pdf.
